# In situ topographical chemical and electrical imaging of carboxyl graphene oxide at the nanoscale

**DOI:** 10.1038/s41467-018-05307-0

**Published:** 2018-07-23

**Authors:** Weitao Su, Naresh Kumar, Andrey Krayev, Marc Chaigneau

**Affiliations:** 1 College of Materials and Environmental Engineering, Hangzhou Dianzi University, 310018 Hangzhou, China; 2 Key Laboratory of RF Circuits and Systems (Hangzhou Dianzi University), Ministry of Education, Hangzhou, 310018 China; 3 National Physical Laboratory, Hampton Road, Teddington, Middlesex TW11 0LW UK; 4 Inorganic Chemistry and Catalysis group, Debye Institute for Nanomaterials Science, Utrecht University, Universiteitsweg 99, 3584 CG Utrecht, The Netherlands; 5 HORIBA Instruments Incorporated, Novato, CA 94949 USA; 6 HORIBA France, Avenue de la Vauve, Passage Jobin Yvon, 91120 Palaiseau, France

## Abstract

Visualising the distribution of structural defects and functional groups present on the surface of two-dimensional (2D) materials such as graphene oxide challenges the sensitivity and spatial resolution of the most advanced analytical techniques. Here we demonstrate mapping of functional groups on a carboxyl-modified graphene oxide (GO–COOH) surface with a spatial resolution of ≈10 nm using tip-enhanced Raman spectroscopy (TERS). Furthermore, we extend the capability of TERS by measuring local electronic properties in situ, in addition to the surface topography and chemical composition. Our results reveal that the Fermi level at the GO–COOH surface decreases as the *I*_D_/*I*_G_ ratio increases, correlating the local defect density with the Fermi level at nanometre length-scales. The in situ multi-parameter microscopy demonstrated in this work significantly improves the accuracy of nanoscale surface characterisation, eliminates measurement artefacts, and opens up the possibilities for characterising optoelectronic devices based on 2D materials under operational conditions.

## Abstract (web summary)

Mapping the distribution of functional groups on 2D materials with high resolution remains challenging. Here, the authors combine tip-enhanced Raman spectroscopy and Kelvin probe force microscopy to simultaneously examine the topography, chemical composition and electronic nature of graphene oxide surfaces with nanoscale spatial resolution.

## Introduction

In recent years, graphene oxide (GO) has attracted much attention in the field of two-dimensional (2D) materials research because of its many potential applications, including lithium batteries^1^, energy harvesting^2,3^ and novel medicines^4^. A GO flake typically comprises a few-nanometre thick sheet of carbon atoms bonded to functional groups, such as carboxyl, carbonyl, hydroxyl or nitro^5–7^. These functional groups can be further linked to RNA-aptamers^8^ or even peptides^9^ resulting in functionalised GO^6^ with fascinating tunable properties. However, visualising the distribution of functional groups on a GO surface at the nanoscale challenges the chemical sensitivity and spatial resolution of most conventional analytical techniques, such as X-ray photoelectron spectroscopy (XPS) (millimetre spatial resolution)^7,10^, confocal Raman spectroscopy (200–300 nm spatial resolution)^7,10^ and aberration-corrected transmission electron microscopy (TEM) (limited molecular information)^11^.

Tip-enhanced Raman spectroscopy (TERS) has emerged as a powerful analytical technique providing high chemical sensitivity for surface molecular mapping with nanoscale spatial resolution under ambient conditions^12–14^. In TERS, a metallic scanning probe microscopy (SPM) probe placed at the focal point of a laser undergoes localised surface plasmon resonance (LSPR), which together with the lightening rod effect leads to the enhancement and confinement of the electric field at the TERS probe apex. This effect simultaneously improves the sensitivity as well as the spatial resolution of Raman microscopy by enhancing the Raman signal from analyte molecules directly underneath the TERS probe. In previous reports, TERS has been successfully used to characterise ultrathin organic films^15^, single molecules^16^ and various material properties of graphene, such as the number of layers^17–19^, local strain^20^, edge orientation^14,21^, surface adsorbates^13,22^ and artificial defects^23^. However, to the best of our knowledge, the capability of TERS to detect functional groups on GO or a functionalised GO surface has not been demonstrated^13,17,18,24^.

Herein, we present nanoscale surface mapping of structural defects and functional groups on few-layer carboxyl-modified graphene oxide (GO–COOH) flakes with ≈10 nm spatial resolution using TERS. Furthermore, we take the surface characterisation a step further by combining TERS with Kelvin probe force microscopy (KPFM) and demonstrate in situ topographical, chemical and electrical nanoscopy of a GO–COOH surface. These measurements reveal an inverse correlation between the local defect density and contact potential difference (CPD) at GO–COOH surface. This work paves the way for optimisation of optoelectronic devices based on 2D materials via simultaneous, non-destructive and multi-parameter characterisation of their surface properties in situ.

## Results

### High-resolution TERS mapping of GO–COOH

Microchemical analysis of the GO–COOH sample used in this study was carried out using XPS (Supplementary Fig. 1), Fourier-transform infrared (FTIR) spectroscopy (Supplementary Fig. 2a) and Raman spectroscopy (Supplementary Fig. 2b). The XPS binding energies and the fingerprint FTIR absorption bands indicate that the GO–COOH sample consists of mainly carbon and oxygen, along with the C–O–C, C–O, C–CH_3_, C=O, COOH and C–H functional groups. See Supplementary Note 1 for details.

**Fig. 1:**
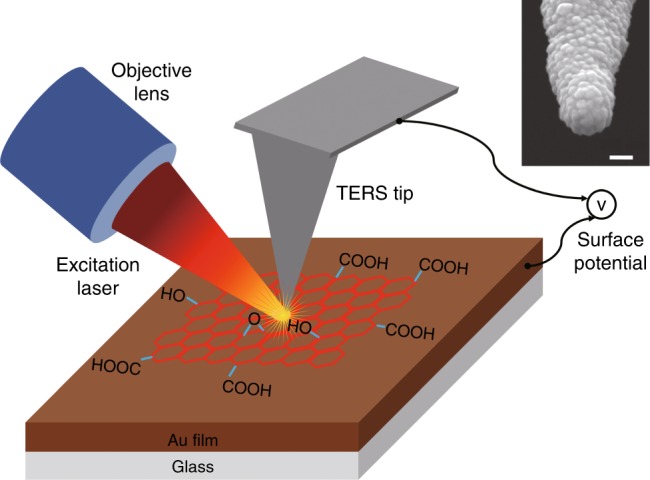
Schematic diagram of the experimental setup. The side illumination AFM-TERS setup used in this work. Au coated AFM probe in combination with 638 nm excitation laser is used for in situ topographical, chemical and electrical nanoscopy of a GO–COOH sample. Scanning electron microscopy (SEM) image of a representative Au coated TERS probe is shown inset. Scale bar: 100 nm

**Fig. 2:**
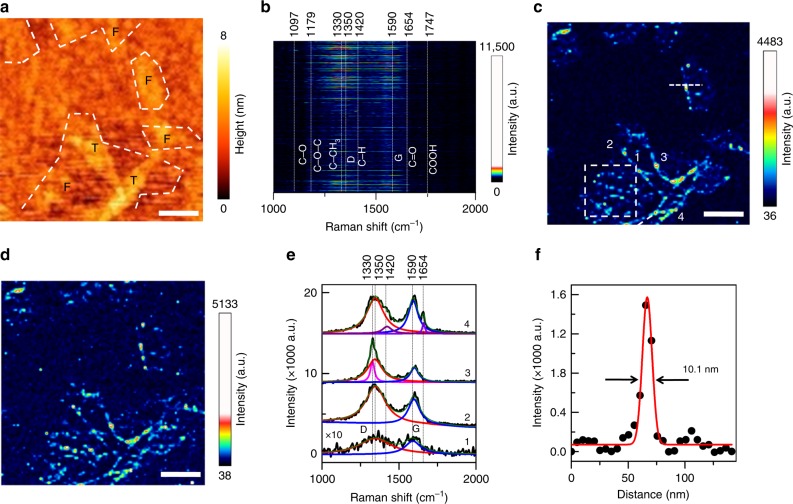
High-resolution TERS mapping of a GO–COOH flake. **a** Topography map of a multilayer GO–COOH sample obtained whilst TERS mapping. “T” and “F” refer to thick-layer and few-layer GO–COOH flakes, respectively. **b** Image of the stacked TERS spectra measured from 100 × 100 pixels across the GO–COOH sample area shown in (**a**). TERS spectra are stacked in the order of their acquisition with the 1st and the 10,000th TERS spectrum presented at the bottom and top of the image, respectively. TERS maps of **c** D band (1350 cm^−1^) intensity and **d** G band (1590 cm^−1^) intensity measured from the GO–COOH sample area shown in (**a**). Nominal pixel dimension: 10 nm. Integration time: 0.4 s. **e** TERS spectra measured at the locations marked 1–4 in (**c**) along with the fitted Lorentzian curves. The intensity of the averaged TERS spectrum from location 1 has been multiplied by 10 for easier visualisation. **f** TERS intensity profile along the white dashed line marked in (**c**) fitted with a Gaussian curve. All scale bars: 200 nm

A schematic diagram of the reflection mode atomic force microscopy (AFM)-TERS setup used in this work is shown in Fig. 1. Figure 2a shows the topography map of a GO–COOH sample containing multilayer flakes, which was recorded during the TERS mapping measurement. A height profile across the topography map shown in Supplementary Fig. 3 indicates that the sample contains both few-layer (1–2 layers) and thick-layer (about 5 layers) GO–COOH flakes. The spatial resolution of this topography map is relatively poor due to the large radius (≈100 nm) of the Au coated TERS probe apex (inset of Fig. 1) compared to typical AFM probes that typically have an apex radius of <10 nm^14^. High-resolution TERS mapping was conducted on the GO–COOH sample area shown in Fig. 2a. The stacked TERS spectra measured at each pixel in the TERS map are shown in Fig. 2b. These stacked TERS spectra exhibit graphene D and G bands, which arise from the scattering by a defect phonon and the first-order scattering by the E_2g_ phonon, respectively^25^, along with the Raman bands corresponding to the functional groups observed in the XPS and FTIR measurements presented in Supplementary Figs. 1 and 2, respectively. Assignment of the Raman bands of six functional groups observed in the TERS spectra along with the D and G bands is presented in Table 1. See Supplementary Note 1 and 2 for details about the assignment of these bands.

**Table 1:** Proposed assignment of Raman bands observed in the TERS spectra in Fig. 2b

Raman band position (cm^−1^)	Tentative assignment
1097	C–O^30,32^ (*ν*_s, C–O_)
1179	C–O–C^30,32^ (*ν*_s, C–O–C_)
1330	C–CH_3_^30,32^ (*δ*_s, C–CH__3_)
1350	D band (GO)^29^
1420	C–H^30,32^ (*β*_C–H_)
1590	G band (GO)^29^
1654	C=O^30,32^ (*ν*_s, C=O_)
1747	COOH^30,32^ (*ν*_s, COOH_)

*ν*_s_ symmetric stretching mode, *β* bending mode, *δ*_s_ symmetric deformation mode

Each spectrum in the TERS map was fitted using eight Lorentzian curves at the band positions listed in Table 1 and eight TERS maps were generated. The TERS intensity maps of the D and G bands are shown in Fig. 2c, d, respectively. In these TERS maps, big (≈1 µm) and small (100-300 nm) GO–COOH flakes can be clearly visualised. The edges and steps of different flakes in the TERS maps are much clearer than in the corresponding topography map shown in Fig. 2a indicating a higher TERS signal enhancement at these locations. The most likely cause of the higher signal enhancement at the steps and edges is the disruption of the GO–COOH lattice leading to a better alignment of the Raman polarisability tensor with the axial polarisation of the tip-enhanced near-field resulting from the gap-mode configuration^16,26–28^. Furthermore, high TERS signal enhancement for the D and G bands is restricted to a few pixels at the steps and edges of the GO–COOH flakes confirming the high spatial resolution of these TERS maps.

 During TERS mapping, markedly different spectra were measured in different areas of the GO–COOH flakes indicating the heterogeneity of surface functionalisation and non-uniform enhancement of the D and G bands. Fig. 2e shows four representative TERS spectra measured at the locations marked 1–4 in Fig. 2c. The TERS spectrum of location 1 represents an averaged spectrum of 10 pixels from a low intensity region. This spectrum contains pronounced Raman bands around 1350 cm^−1^ and 1590 cm^−1^ correlating with the D and G band positions of GO–COOH^29^, confirming that this region contains the GO–COOH lattice. The TERS spectrum at location 2 is similar to that of location 1; although, the signal intensity is around 20 times stronger. However, the TERS spectrum at location 3 shows a very sharp and asymmetric D band and an additional Raman band at 1330 cm^−1^ overlaps with the D band, which could be attributed to the symmetric deformation mode (*δ*_s, C–CH__3_) of C–CH_3_^30,31^. In the TERS spectrum for location 4, two new Raman bands are observed at 1654 cm^−1^ and 1420 cm^−1^, which could be assigned to the symmetric stretching mode (*ν*_s, C=O_) of C=O^32^ and bending mode (*β*_C-H_) of C–H^30^, respectively.

To investigate the distribution of functional groups on the GO–COOH surface, we generated TERS intensity maps of the Raman bands corresponding to six different functional groups (Table 1), which are presented in Supplementary Fig. 4a–f. In these maps, a heterogeneous distribution of different functional groups is observed on the GO–COOH surface (see Supplementary Note 2 for details). TERS maps of the 1097 cm^−1^, 1179 cm^−1^, 1654 cm^−1^, and 1747 cm^−1^ bands shown in Supplementary Fig. 4a, b, e, f, respectively indicate that the C–O, C–O–C, C=O and COOH groups are present only at a few locations on the GO–COOH surface. Furthermore, these functional groups are distributed over the entire GO–COOH surface. These experimental observations cannot be explained by the Lerf–Klinowski theoretical model of GO, according to which, COOH should be located on the edges, whereas other groups, such as C=O and C–O–C should be distributed over the entire surface^33^. We propose that the discrepancy between the Lerf–Klinowski theoretical model and our experimental results could be caused by the two-step method used in the preparation of the GO–COOH sample, whereby only a fraction of the C–O and C–O–C groups obtained on the GO flake in the first step are converted to COOH in the second step^34^. Further understanding of this discrepancy would require a detailed analysis of the sample preparation process and its influence on the lattice structure of GO–COOH, which is beyond the scope of this study. From the TERS maps, the area percentages of the C–O, C–O–C, C=O and COOH groups on GO–COOH surface are calculated to be 1.3%, 1.0%, 0.5% and 0.6%, respectively. However, in the TERS maps of the 1330 cm^−1^ and 1420 cm^−1^ bands (Supplementary Fig. 4c, d) a stronger TERS intensity is observed at a relatively larger number of areas on the GO–COOH flakes, indicating that the C–CH_3_ and C–H groups have a more dominant presence on the GO–COOH surface. Furthermore, the TERS maps in Supplementary Fig. 4 indicate that the different functional groups are distributed differently over the GO–COOH surface. In particular, in the TERS map of 1330 cm^−1^ band intensity in Supplementary Fig. 4c, C–CH_3_ groups show a clearly higher enhancement at the steps and edges of the GO–COOH flakes, whereas the TERS maps for other functional groups indicate a random distribution over the entire GO–COOH surface (see Supplementary Note 2). On the GO–COOH  sample used in this study, the functional groups having Raman polarisability tensors aligned with the axial polarisation of the tip-enhanced near-field are expected to experience a much higher signal enhancement due to the gap-mode TERS surface selection rule^16,26–28^, rendering them more visible. This suggests that compared to the other functional groups, C–CH_3_ groups likely bind to the steps and edges of GO–COOH in a more well-defined orientation with their Raman polarisability tensor aligned with the near-field axial polarisation.

The spatial resolution of the TERS map in Fig. 2c can be calculated by fitting a line profile across a sharp feature with a Gaussian curve^35–37^. An example of this is shown in Fig. 2f, where the spatial resolution of the TERS map is calculated to be around 10.1 nm, from the FWHM of the Gaussian curve fitted to the line profile. In order to obtain a statistical estimate, we performed Gaussian fitting of 5 line profiles from different locations in the TERS map and calculated the average spatial resolution to be 10.5 ± 1.7 nm (Supplementary Fig. 5). Further discussion of the obtained spatial resolution and comparison with previous studies^28,38–41^ is presented in Supplementary Note 3. To demonstrate the reproducibility of nanoscale spatial resolution we conducted TERS mapping on the same GO–COOH flakes using three different TERS tips, the results of which are presented in Supplementary Fig. 8. All three TERS tips provided a similar spatial resolution in the TERS maps (comparable to the step size) and displayed reproducibility of chemical imaging as discussed in Supplementary Note 4.

### Visualising structural defects and functional groups

**Fig. 3:**
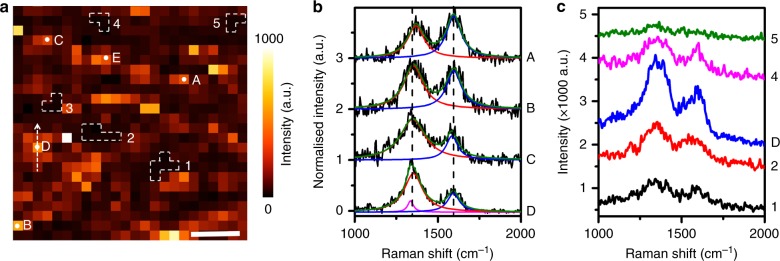
Visualising structural defects and functional groups on the GO–COOH surface at the nanoscale. **a** Zoomed-in TERS map of D band intensity from 250 × 250 nm^2^ area marked with a dashed square in Fig. 2c. Scale bar: 50 nm. **b** TERS spectra measured at the locations marked as A–D in (**a**). **c** TERS spectra measured at five pixels along the arrow marked across location D in (**a**). Note that the area of the TERS map in (**a**) is only around 0.5 % of the diffraction limited laser spot area for our confocal Raman microscope

To demonstrate the capability of TERS for providing rich chemical information with a high spatial resolution, we present further analysis of the TERS map shown in Fig. 2c. A zoomed-in TERS map of D band intensity from the area marked by the dotted square in Fig. 2c is presented in Fig. 3a. In this TERS map, areas with high TERS D band intensity (>900 counts) are observed interspersed with several areas having a low signal intensity (30-40 counts), which is close to the noise level. Averaged TERS spectra from five low intensity areas marked using white dashed lines in Fig. 3a are presented in Supplementary Fig. 9 (see Supplementary Note 5 for details). In the averaged spectra measured at these locations, no D or G bands are observed indicating a discontinuity in the GO–COOH lattice. Previously, using TEM, Erickson et al. have shown that few-layer GO–COOH flakes are composed of functionalised areas with large vacancies inside^11^. Therefore, these low signal intensity areas with unobservable D and G bands can be attributed to the vacancies present within the GO lattice. From inspection of individual TERS spectra, the size of these vacancies was determined to be 100–600 nm^2^ (1–6 pixels) correlating well with the size reported by Erickson et al.^11^.

Furthermore, in the TERS map, remarkably different spectra were observed at different areas with high signal intensity. 38 TERS spectra from areas of high-signal intensity in Fig. 3a are plotted in Supplementary Fig. 10. The ratio of D to G band intensity (*I*_D_/*I*_G_) is commonly used to quantify defect density in graphene^42^ and GO^10^. The 38 spectra can be broadly classified into two groups. The first group (Supplementary Fig. 10b– e) shows only the D and G bands, whereas the second group (Supplementary Fig. 10f) shows Raman bands from multiple functional groups spectrally overlapping with the D and G bands. Based on the *I*_D_/*I*_G_ ratio, the TERS spectra in the first group can be further divided into four types: (1) *I*_D_/*I*_G _< 1 (Supplementary Fig. 10b), (2)* I*_D_/*I*_G_ ≈ 1 (Supplementary Fig. 10c), (3) 1 < *I*_D_/*I*_G _< 1.5 (Supplementary Fig. 10d), (4) *I*_D_/*I*_G _> 1.5 (Supplementary Fig. 10e). Four representative TERS spectra from Supplementary Fig. 10b– e measured at locations A–D in Fig. 3a, are presented in Fig. 3b. In the TERS spectra from A–C, the shape of D and G bands at 1350 cm^−1^ and 1590 cm^−1^ resembles the TERS spectrum at position 2 in Fig. 2e, which can be fitted using a pair of Lorentzian curves. Therefore, spectra measured at A–C can be assigned to GO–COOH areas with increasing density of defects (A < B < C)^10^. The TERS spectrum at location D also shows two Raman bands; however, the Raman band at 1350 cm^−1^ is unusually sharp and more intense than that of Raman band at 1590 cm^−1^ and  cannot be fitted using a single Lorentzian curve. This spectrum is similar to the TERS spectrum at position 3 shown in Fig. 2e, and therefore, can be assigned to a combination of the D band and *δ*_s, C–CH__3_ vibrational mode of C–CH_3_^30,31^. In contrast to TERS spectra of the first group (showing only D and G band features), TERS spectra of the second group (location E) shown in Supplementary Fig. 10f exhibit a number of different Raman bands. The positions of these Raman bands correlate well with the bands listed in Table 1 indicating that they represent different functional groups present on the GO–COOH surface. It should be noted that the proportions of the two groups of TERS spectra shown in Supplementary Fig. 10 are quite different: 85% of the spectra are in first group (positions A–D), whereas only 15% spectra are in the second group (position E) as indicated by the histogram shown in Supplementary Fig. 11. This indicates that the surface of this GO–COOH sample is only partially functionalised.

Interestingly, the TERS spectra measured from pixels in the immediate vicinity of positions A–E were found to be similar to each other. The TERS spectra from five pixels along the line marked across position D in Fig. 3a are plotted in Fig. 3c. In order to eliminate the possibility of artefacts caused by occasional contamination of TERS tip, these lines were chosen normal to the scan direction of TERS mapping. All TERS spectra shown in Fig. 3c exhibit typical broad Raman bands at ≈1350 cm^−1^ and ≈1590 cm^−1^, the positions of D and G bands. *I*_D_/*I*_G_ is calculated to be 1.8 for position D, whereas 20 nm away from position D it decreases to 1.2 (Supplementary Fig. 12). Therefore, the pixels across position D represent a cluster of defects with the highest defect density at the center and decreasing away from it. This reveals heterogeneity in the distribution of defects on the GO–COOH surface within the length-scale of a few nanometres. To the best of our knowledge, such high-resolution chemical information from a GO–COOH surface cannot be obtained non-destructively using any other analytical technique.

### In situ topographical chemical and electrical nanoscopy

**Fig. 4:**
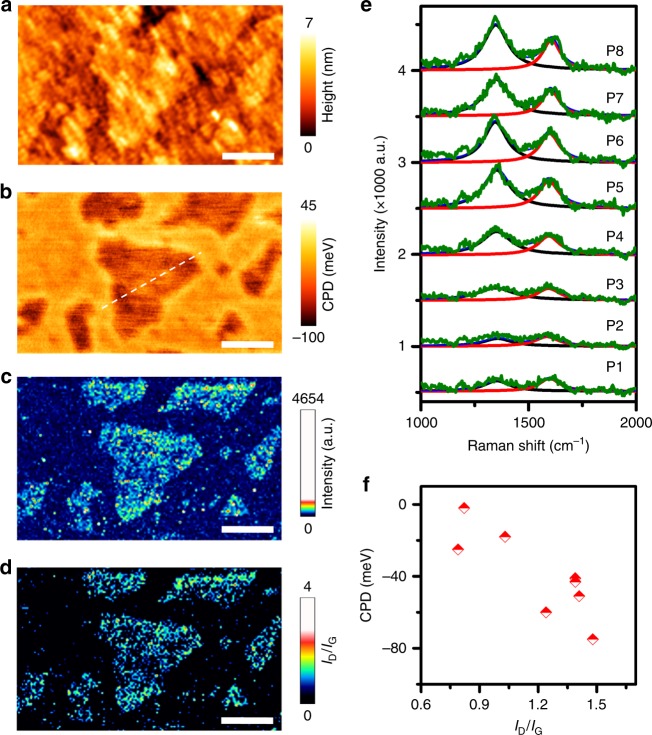
In situ topographical, chemical and electrical nanoscopy of GO–COOH. **a** Topography map of a few-layer GO–COOH sample obtained whilst TERS mapping. **b** CPD map, **c** TERS map of D band (1350 cm^−1^) intensity and **d** *I*_D_/*I*_G_ ratio map measured in the region shown in (**a**). Area: 2.5 × 1.7 µm^2^. Step size: 16.7 nm. Integration time: 75 ms/pixel. **e** Averaged TERS spectra from the 8 different locations marked as P1–P8 in Supplementary Fig. 15 fitted with two Lorentzian curves. **f** Plot of CPD as a function of *I*_D_/*I*_G_ ratio calculated from the averaged TERS spectra shown in (**e**) showing an inverse correlation between local defect density and Fermi level on the GO–COOH surface. All scale bars: 500 nm

To study the variation of local electronic properties due to functional groups present on the GO–COOH surface, we carried out in situ topographical, chemical and electrical nanoscopy by performing KPFM & TERS measurements consecutively in the same sample area using the same Au coated TERS tip. Topographic image of a few-layer GO–COOH sample obtained whilst TERS mapping is shown in Fig. 4a. This topography map has a poor spatial resolution because of the relatively large size of the TERS tip-apex, but shows several few-layer GO–COOH flakes (1–2 nm thickness) along with a ≈1 µm size GO–COOH flake at the centre. A contact potential difference (CPD) map measured in this region using KPFM is shown in Fig. 4b. In this map, 200–500 nm size areas with a significantly different CPD are observed within the GO–COOH flakes. A line profile across the central GO–COOH flake in Fig. 4b presented in Supplementary Fig. 13 reveals CPD variation of −87 to 34 meV. Since the Fermi level of gold (*E*_f_Au_) is 5.1 eV^43,44^ and the Fermi level of GO–COOH (*E*_f_GO_) is equal to *E*_f_Au_ + CPD, *E*_f_GO_ is found to vary from 5.01 to 5.13 eV across the central GO–COOH flake. This Fermi level is similar to the previously reported value of 5 eV of GO^45^, but is higher than the value of 4.5 eV for graphene^43,44^.

After KPFM measurements, we performed in situ TERS measurement in the same sample area shown in Fig. 4a, b. TERS maps of D and G band intensities at 1350 cm^−1^ and 1590 cm^−1^ are shown in Fig. 4c and Supplementary Fig. 14, respectively. The TERS *I*_D_/*I*_G_ ratio image is shown in Fig. 4d. In these TERS maps, a heterogeneous distribution of D and G band intensity is observed across all GO–COOH flakes. The TERS intensity variations indicate local differences in surface chemistry, which is expected to cause variation of surface potential over GO–COOH flakes. To probe correlation of local defect density and surface potential, we calculated the *I*_D_/*I*_G_ ratio from the TERS spectra averaged over an area of 0.012 µm^2^ (50 pixels) from 8 different locations on the GO–COOH flake as shown in Supplementary Note 6. The averaged TERS spectra are shown in Fig. 4e. The *I*_D_/*I*_G_ ratio calculated from the averaging of TERS spectra over a large area should be similar to the far-field measurement, which is conventionally used to characterise the defect level in graphene^42^ and graphene oxide^10^, thereby allowing comparison of the CPD with the local defect density of the GO–COOH flake despite different spatial resolutions of the TERS and CPD maps. The *I*_D_/*I*_G_ ratio was calculated by fitting the averaged TERS spectra using two Lorentzian curves at 1350 cm^−1^ and 1590 cm^−1^. A plot of the CPD versus *I*_D_/*I*_G_ ratio from the eight areas is shown in Fig. 4f indicating an inverse correlation between the two parameters. A high CPD (≈ −20 meV, Fermi level = 5.08 eV) is observed in the areas (P1, P2) with *I*_D_/*I*_G _< 1, whereas a low CPD (≈ −80 meV, Fermi level = 5.02 eV) is observed in the areas (P6, P7, P8) with *I*_D_/*I*_G_ > 1.3. Interestingly, this inverse correlation of Fermi level with *I*_D_/*I*_G_ ratio on the GO–COOH surface observed in this work is different to that of α-beam irradiated graphene, where the Fermi level has been reported to increase from 4.5 to 4.9 eV with increasing density of defects^44^. This difference could result from the presence of different kinds of defects on GO–COOH surface. These results demonstrate that the in situ KPFM and TERS method allows a direct correlation of the Fermi level of a GO–COOH flake to the defect density at the nanoscale, thereby significantly improving the accuracy of GO–COOH surface characterisation.

In summary, we have demonstrated nanoscale TERS mapping of structural defects and functional groups present on a GO–COOH surface with an unprecedented spatial resolution of ≈10 nm. Furthermore, we have pushed nanoscale surface characterisation a step forwards by demonstrating in situ topographical, electrical and chemical nanoscopy of a GO–COOH sample by combining TERS with KPFM. This in situ multi-parameter measurement methodology greatly extends the capability of TERS allowing a direct correlation of local topography, chemical composition and electronic properties at the nanoscale not only in 2D materials but on almost any sample surface. In particular, we expect this work to open up the possibility of optimising optoelectronic devices based on novel 2D materials such as graphene, GO, single-layer MoS_2_ and others via non-destructive, simultaneous and nanoscale multi-parameter characterisation of their surface properties even under operational conditions.

## Methods

### TERS measurements

TERS measurements were performed on a NanoRaman system consisting of an atomic force microscope (OmegaScope, formerly AIST-NT, now HORIBA Scientific) combined with a Raman spectrometer (XploRA, HORIBA Scientific, France) in side illumination geometry as schematically shown in Fig. 1. A 638 nm excitation laser was focussed on the sample at an angle of 60° using a ×100, 0.7 NA, 20 mm long working distance objective lens (Mitutoyo, Japan). TERS spectra were measured using a spectrometer grating with 600 lines/mm and an electron-multiplying charged-coupled device detector (Andor, Ireland) with a laser power of 100 µW at the sample.

For fast and efficient TERS mapping, TERS measurements were conducted in SpecTop™ TERS mapping mode in which TERS spectrum at a particular pixel in the TERS map is measured when the tip is in direct contact with the surface, with a typical interaction force of 2–10 nN and integration time of 0.05 s–0.5 s. Transition between the pixels of the TERS map is performed in semi-contact mode, which preserves both the sharpness and plasmonic enhancement of the tip eliminating lateral forces that might otherwise sweep aside or pick up loosely attached contaminants from the sample surface. All TERS measurements were performed using Au coated AFM TERS tips (*k* = 7 N/m, *f* = 150 kHz, formerly AIST-NT, now HORIBA Scientific).

### Sample preparation

GO–COOH samples for TERS measurements were prepared by spin-coating GO–COOH (ACS Material, USA) on a Au coated glass substrate.

### Data availability

The data supporting the findings of this study are available from the corresponding authors upon request.

## Electronic supplementary material

Supplementary Information
