# Supplementary material for: In situ topographical chemical and electrical imaging of carboxyl graphene oxide at the nanoscale
Source: Nat Commun. 2018 Jul 23;9:2891. doi: 10.1038/s41467-018-05307-0 (PMC6056528; doi:10.1038/s41467-018-05307-0)
Supplement: Supplementary file 1 — Supplementary Information [file 41467_2018_5307_MOESM1_ESM.pdf]

Supplementary Information

In Situ Topographical Chemical and Electrical Imaging of Carboxyl Graphene Oxide at the Nanoscale

Weitao Su *et al.*

In Situ Topographical Chemical and Electrical Imaging of Carboxyl Graphene Oxide at the Nanoscale

Weitao Su[1,2], Naresh Kumar[3,4], Andrey Krayev[5] and Marc Chaigneau[6]

[1]College of Materials and Environmental Engineering, Hangzhou Dianzi University, 310018, Hangzhou, China

[2] Key Laboratory of RF Circuits and Systems, Hangzhou Dianzi University, Ministry of Education, Hangzhou, 310018, China

[3]National Physical Laboratory, Hampton Road, Teddington, Middlesex, TW11 0LW, UK

[4]Inorganic Chemistry and Catalysis group, Debye Institute for Nanomaterials Science, Utrecht University, Universiteitsweg 99, 3584 CG Utrecht, The Netherlands

[5]HORIBA Instruments Incorporated, Novato, California 94949, United States

[6]HORIBA France, Avenue de la Vauve, Passage Jobin Yvon, 91120 Palaiseau, France

Correspondence and requests for materials should be addressed to W.S., N.K. or M.C. (email: suweitao@hdu.edu.cn, naresh.kumar@npl.co.uk, Marc.CHAIGNEAU@horiba.com)

Supplementary Note 1: Chemical characterisation of the GO-COOH sample

The carboxyl-modified graphene oxide (GO-COOH) sample used in this study was obtained from ACS Material LLC (USA)[1]. This material has been used in several previous studies of GO-COOH[2, 3]. Herein, we present chemical characterisation of this sample using X-ray photoelectron spectroscopy (XPS), Fourier-transform infrared spectroscopy (FTIR) and Raman spectroscopy.

The XPS survey spectrum of the GO-COOH sample is presented in Supplementary Fig. 1a. In this spectrum, the dominant intensity of C1s and O1s bands indicates that the GO-COOH sample mainly consists of carbon and oxygen, whereas the concentration of nitrogen and sulfur is negligible. The C / O / N / S atomic ratio is calculated to be 67.1 % / 31.9 % / 0.5 % / 0.5 %. Due to instrumental limitations, the concentration of hydrogen could not be measured[4]. The C1s band can be further deconvoluted into sp^2 carbon (284.4 eV), sp^3 carbon (285.2 eV), C-O (286.7 eV, from C-O or C-O-C), C=O (287.9 eV) and COOH (289.3 eV) bands[4]. The O1s bands can be deconvoluted into C=O (531.3 eV), C-O (532.5 eV) and H_2O (533.8 eV) bands[4]. The deconvolution of the C1s and O1s spectra correlates well with the vibrational modes identified in the FTIR absorption and Raman spectra of GO-COOH as shown below.

FTIR and Raman measurements of the GO-COOH sample are presented in Supplementary Fig. 2. In the FTIR absorption spectrum (Supplementary Fig. 2a), the broad band in the 3000 cm^{-1} – 3500 cm^{-1} region can be assigned to H_2O or the stretching mode of O-H ($\nu_{S, O\text{-}H}$) from C-OH, COOH[5,6]. The double wavelet bands at 2864 cm^{-1} and 2924 cm^{-1} can

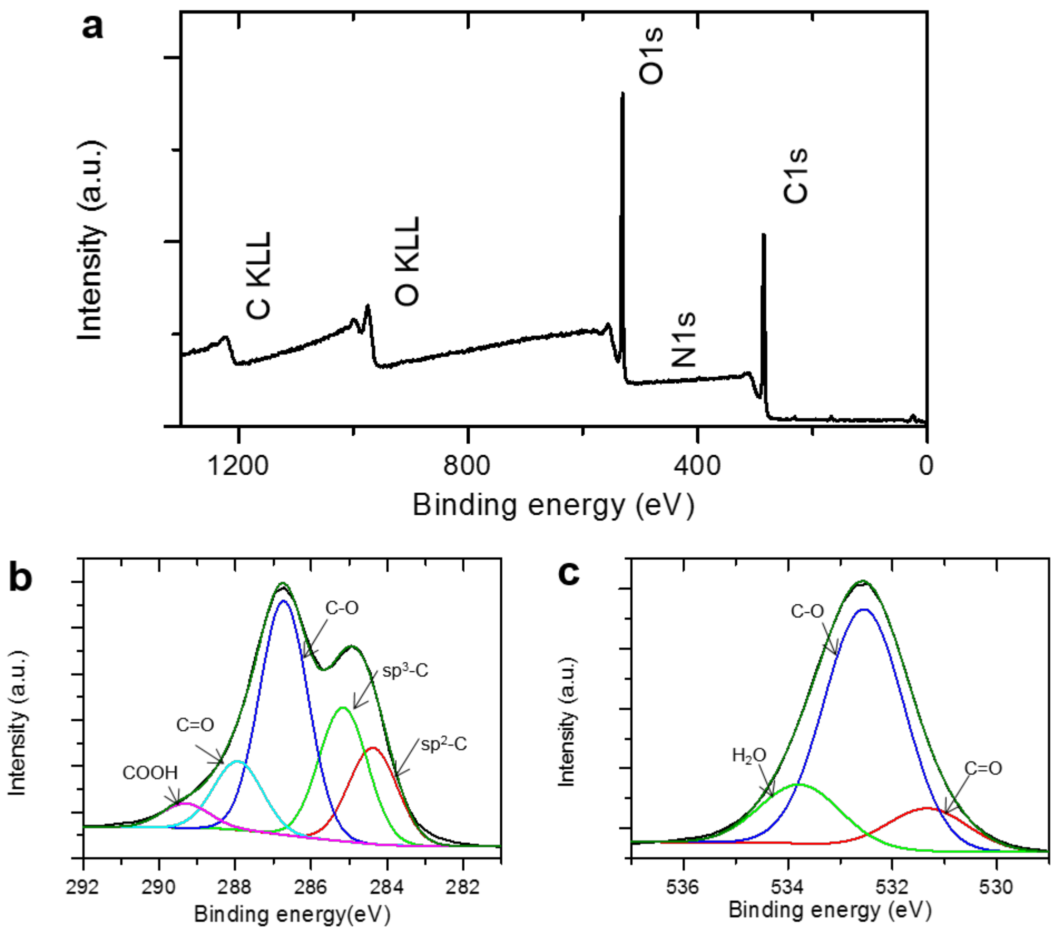

Supplementary Fig. 1 **a** XPS survey spectrum of the GO-COOH sample. XPS **b** C1s and **c** O1s spectra of the GO-COOH sample.

be assigned to symmetric ($v_{S,\ C\text{-}H}$) and asymmetric ($v_{as,\ C\text{-}H}$) stretching modes of C-H, respectively[7]. The bands at 1721 cm^{-1}, 1607 cm^{-1}, 1391 cm^{-1}, 1073 cm^{-1}, 869 cm^{-1} and 1186 cm^{-1} can be assigned to the stretching mode of COOH ($v_{S,\ COOH}$)[5,6], stretching mode of C=O ($v_{S,\ C=O}$)[5,6], C-C stretching of C-CH_3 ($v_{S,\ C\text{-}C}$)[8], stretching mode of C-O ($v_{S,\ C\text{-}O}$)[5,6], asymmetric ($v_{as,\ C\text{-}O\text{-}C}$) and symmetric stretching ($v_{s.\ C\text{-}O\text{-}C}$) of C-O-C[5,6], respectively.

In the Raman spectrum of the GO-COOH flake (Supplementary Fig. 2b) prominent bands are observed at 1352 cm^{-1} and 1583 cm^{-1}, which are assigned to the D and G bands of GO-COOH[9]. A very weak 2D band[10] is observed at ≈2700 cm^{-1}. The broad band in the 2900 cm^{-1} - 3000 cm^{-1} region can be assigned to the stretching mode of C-H ($v_{S,\ C\text{-}H}$)[11]. Because the E_{2g} phonon is infrared (IR) inactive, this mode cannot be observed in the FTIR absorption spectrum[12]. Moreover, D and 2D Raman vibrational modes are also IR inactive, and hence cannot be observed in the FTIR spectrum[13]. In the Raman spectrum of GO-COOH, the very strong intensity of D and G bands makes the observation of Raman bands of other functional groups (which are present in relatively small concentration) difficult. Note that in the TERS maps measured from this sample (Fig. 2 and 4), due to the short integration time of 0.1 s - 0.4 s used at each pixel, the weak 2D band is immersed in the background noise and hence is not observed.

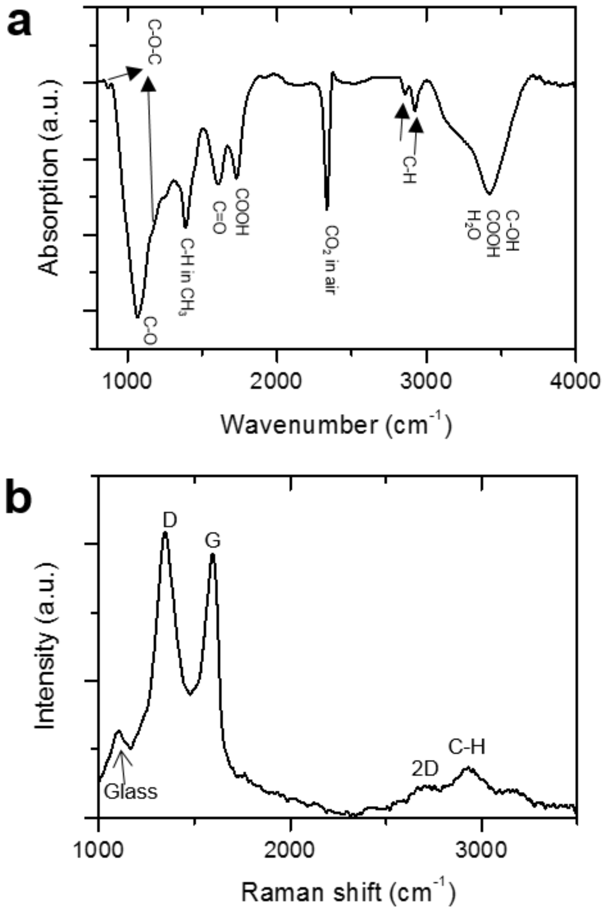

Supplementary Fig. 2 **a** FTIR spectrum measured from the GO-COOH sample. Spectral resolution: 4 cm^{-1}. Integration time: 20 s. **b** Raman spectrum measured from the GO-COOH sample. Integration time: 120 s, Excitation laser: 532 nm, Laser power: 0.1 mW.

The XPS, FTIR and Raman characterisation results are summarised in Supplementary Table 1 below.

Supplementary Table 1. Summary of GO-COOH sample characterisation using XPS, FTIR and Raman spectroscopy.

Proposed assignment of functional groups	XPS binding energy (eV)	FTIR bands (cm^{-1})	Raman bands (cm^{-1})
O-H	533.8[4]	3000-3500[5,6]	-
C-H	285.2[4]	2864, 2924[7]	2900[11]
COOH	289.3, 532.5[4]	1721[5,6]	-
C=O	287.9, 531.3[4]	1607[5,6]	-
CH_3	285.2[4]	1391[8]	
C-O-C	286.7[4]	1186, 869[5,6]	-
C-O	286.7[4]	1073[5,6]	-
D	-	-	1350[9]
G	-	-	1590[9]
2D	-	-	2700[10]

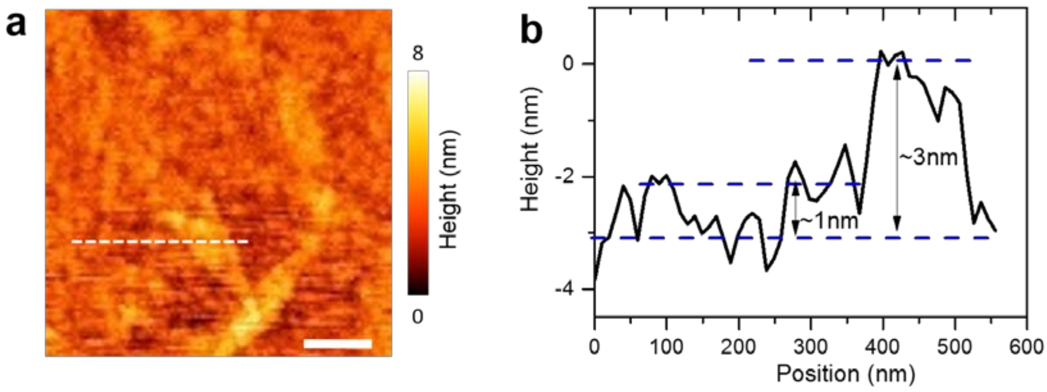

Supplementary Fig. 3 **a** AFM topography image of a multilayer GO-COOH sample (Fig. 2a) recorded whilst TERS mapping. Scale bar: 200 nm. **b** Height profile along the line marked in **a** indicating that the sample contains both few-layer (1-2 layers) and thick-layer (≈5 layers) GO-COOH flakes.

Supplementary Note 2: Chemical analysis of the TERS map

In the fingerprint region of the TERS spectra, the D and G bands of GO-COOH overlap with the Raman bands of other functional groups. This makes unequivocal assignment of Raman bands to different functional groups difficult. However, FTIR spectroscopy presents an effective solution to the problem of identifying functional groups present in the GO-COOH sample. Therefore, we used the following analytical procedure to identify vibrational bands of functional groups in the TERS spectra: (1) Determine the chemical composition and functional groups present in GO-COOH sample using XPS. (2) Confirm functional groups with the corresponding vibrational modes in the FTIR spectrum. (3) Obtain Raman vibrational bands of the functional groups identified using XPS and FTIR from literature and use these to analyse the spectra measured in the TERS maps.

In the FTIR spectrum of GO-COOH presented in Supplementary Fig. 2a, the following groups can be identified in the fingerprint region: COOH (1721 cm^{-1}), C=O (1607 cm^{-1}), CH_3 (1391 cm^{-1}), C-O-C (1186 cm^{-1}) and C-O (1073 cm^{-1}). All these functional groups are Raman-active[14] and can be observed in the TERS spectra. Stacked TERS spectra measured at every pixel in the TERS map are shown in Fig. 2b. In these spectra, the TERS bands at 1097 cm^{-1}, 1179 cm^{-1}, 1654 cm^{-1} and 1747 cm^{-1} correlate well with the Raman vibrational modes of C-O, C-O-C, C=O and COOH, respectively as listed in Table 1. Additionally, we observe bands at 1420 cm^{-1} and 1330 cm^{-1}, which can be assigned to the Raman vibrational modes of C-H and C-CH_3[15, 16]. Furthermore, the assignment of these bands is also consistent with the functional groups expected in the classical model of graphene oxide such as alcohol (C-OH), ketone (C=O), carboxylic acid (COOH), epoxy and ether (C-O-C)[17].

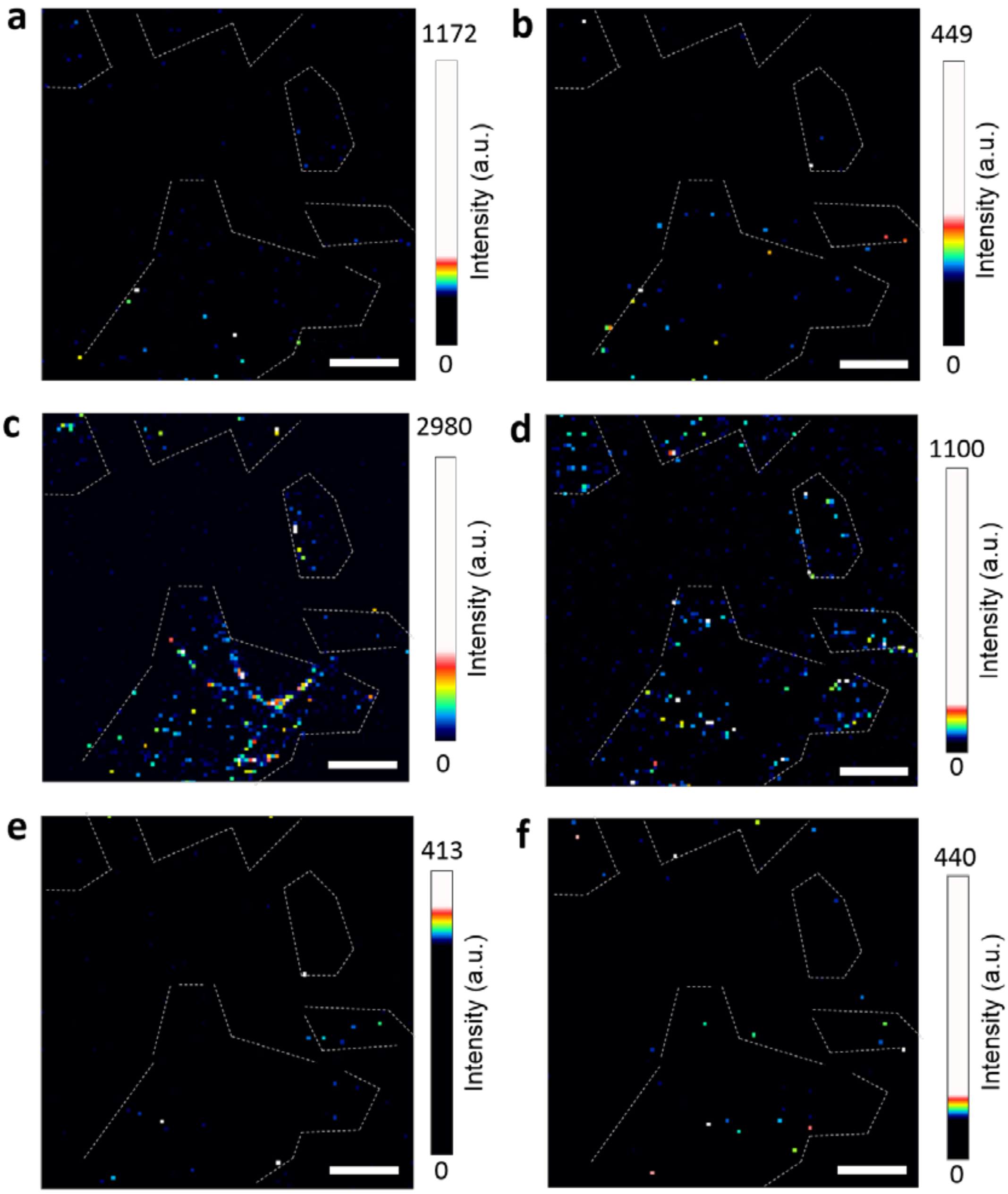

Supplementary Fig. 4 TERS intensity maps of the bands at **a** 1097 cm^{-1} ($\nu_{s, C\text{-}O}$), **b** 1179 cm^{-1} ($\nu_{s, C\text{-}O\text{-}C}$), **c** 1330 cm^{-1} ($\delta_{s, C\text{-}CH3}$), **d** 1420 cm^{-1} ($\beta_{C\text{-}H}$), **e** 1654 cm^{-1} ($\nu_{s, C=O}$) and **f** 1747 cm^{-1} ($\nu_{s, COOH}$). All scare bars: 200 nm.

We fitted each spectrum in the TERS map shown in Figure 2c using 8 different Lorentzian curves at the following positions: 1097 cm^{-1}, 1179 cm^{-1}, 1330 cm^{-1}, 1350 cm^{-1}, 1420 cm^{-1}, 1590 cm^{-1}, 1654 cm^{-1} and 1747 cm^{-1}. In the fitting process, the peak positions of each group were constrained within ± 5 cm^{-1}. The TERS maps of these bands are presented in Fig. 2c - 2d and Supplementary Fig. 4a -4f. In gap mode TERS configuration, the alignment of the Raman polarisability tensor of a molecule with the axial polarisation of TERS near-field causes an extremely high enhancement of the Raman signal[18, 19, 20, 21]. Therefore, we speculate that the Raman signals of the functional groups observed in the TERS maps shown in Supplementary Fig. 4 most likely result from orientational ordering rather than spatial

clustering. A limitation of the present measurements is that it is not possible to determine if the absence of Raman signal at a particular location results from an absence of functional groups or a different orientation. However, this is beyond the scope of the current study and we aim to address it in our future research work.

Supplementary Note 3: Spatial resolution of the TERS map

It should be noted that the spatial resolution of the TERS map obtained in Supplementary Fig. 5 is much smaller than the radius of Au coated TERS tip-apex. The typical size of the Au grains present at the TERS tip-apex ranges from 24 nm – 47 nm as shown in Supplementary Fig. 6, indicating that single Au grains rather than the entire tip-apex is involved in LSPR enhancement of Raman signals[22, 23, 24]. This TERS resolution is 45 × better than the highest confocal spatial resolution that can be achieved in our system, which is calculated using 0.44λ/NA[25] (λ = excitation laser wavelength; NA = numerical aperture of the objective lens) to be 447 nm. Furthermore, this spatial resolution is also higher than the previously reported value for TERS maps of graphene (12 nm - 20 nm)[26] and two-dimensional transition metal dichalcogenides (TMDs) such as MoS_2 (20 nm)[27] and WSe_2

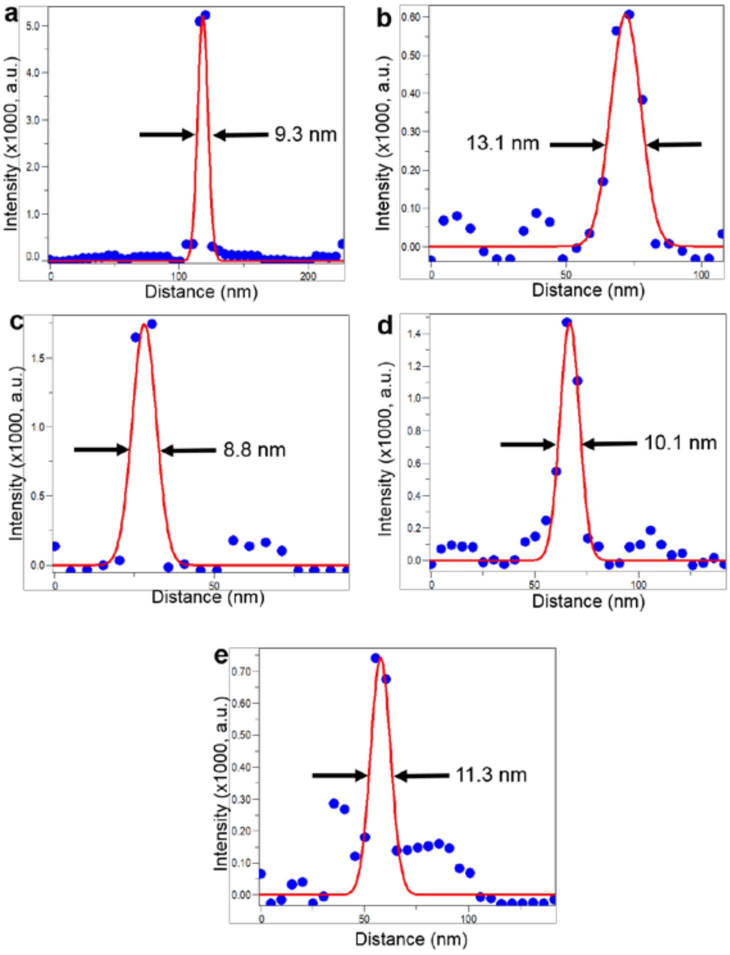

Supplementary Fig. 5 **a** – **e** TERS intensity profiles (blue) along with the fitted Gaussian curves (red) from five different locations in the TERS map shown in Fig. 2c. Average spatial resolution of the TERS map is estimated from the FWHM of the fitted Gaussian curves to be 10.5 ± 1.7 nm.

15 nm)[28] measured using either bottom or side illumination TERS configurations (see Supplementary Fig. 7). The spatial resolution of the TERS map is primarily limited by the size of the plasmonic nanostructures present at the TERS tip-apex. However, we believe that in our TERS measurements the spatial resolution could also be limited by the relatively large step size of 10 nm used for TERS mapping. For example, TERS imaging of 2D polymers with a spatial resolution of < 10 nm was recently reported by Shao *et al.*[18] and Müller *et al.*[29], using a step size of 5 nm. Therefore, TERS mapping with a smaller step size is required to accurately determine the highest spatial resolution possible with the TERS probe used in our measurement.

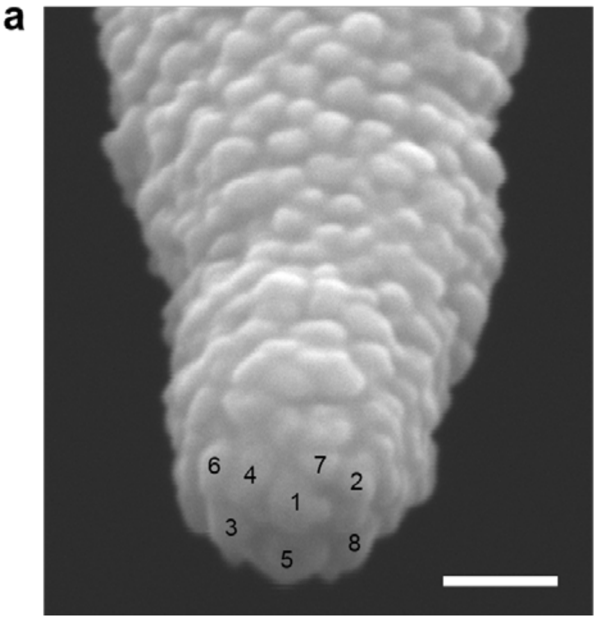

b

Grain position	Grain size (nm)
1	47
2	34
3	38
4	32
5	38
6	24
7	32
8	25

Supplementary Fig. 6 **a** SEM image of a representative Au coated TERS tip used in this work. Scale bar: 100 nm. **b** Table listing the size of the Au grains present at the TERS tip-apex at the locations marked in **a**. The size of the Au grains at the TERS tip-apex ranges from 24 nm – 47 nm.

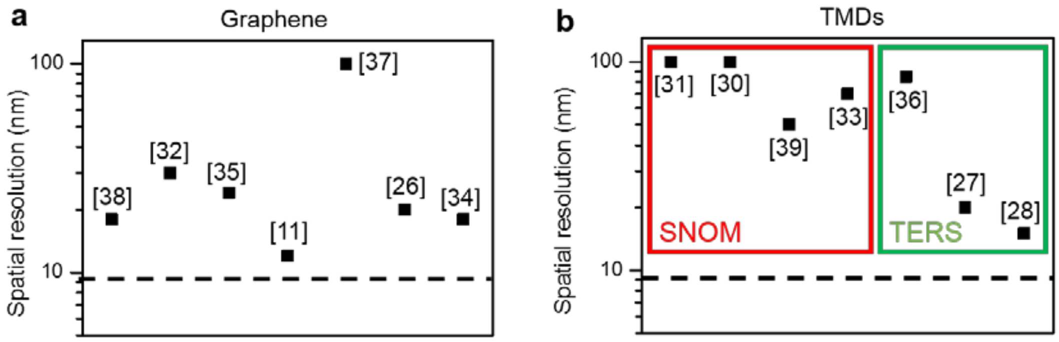

Supplementary Fig. 7 Spatial resolution obtained in TERS images of **a** graphene and **b** TMDs reported in literature[11, 26, 27, 28, 30, 31, 32, 33, 34, 35, 36, 37, 38, 39]. For comparison, the reported spatial resolution of scanning near-field optical microscopy (SNOM) performed on TMDs is also presented in **b**. Black dashed lines marked in **a** and **b** signify the spatial resolution obtained in this work.

Supplementary Note 4: Reproducibility of high-resolution TERS mapping

In Figure 2 and 4, we have shown high-resolution TERS mapping of GO-COOH using two different TERS tips on two different flakes of the same sample. This indicates that different TERS tips can be used to obtain spatially resolved chemical mapping of a GO-COOH sample at the nanoscale. However, in order to further demonstrate the reproducibility of high-resolution TERS mapping, we carried out TERS mapping of the same GO-COOH flake using three different TERS tips. Results of these measurements are presented in Supplementary Fig. 8. Supplementary Fig. 8a shows the AFM topography image of the GO-COOH flake

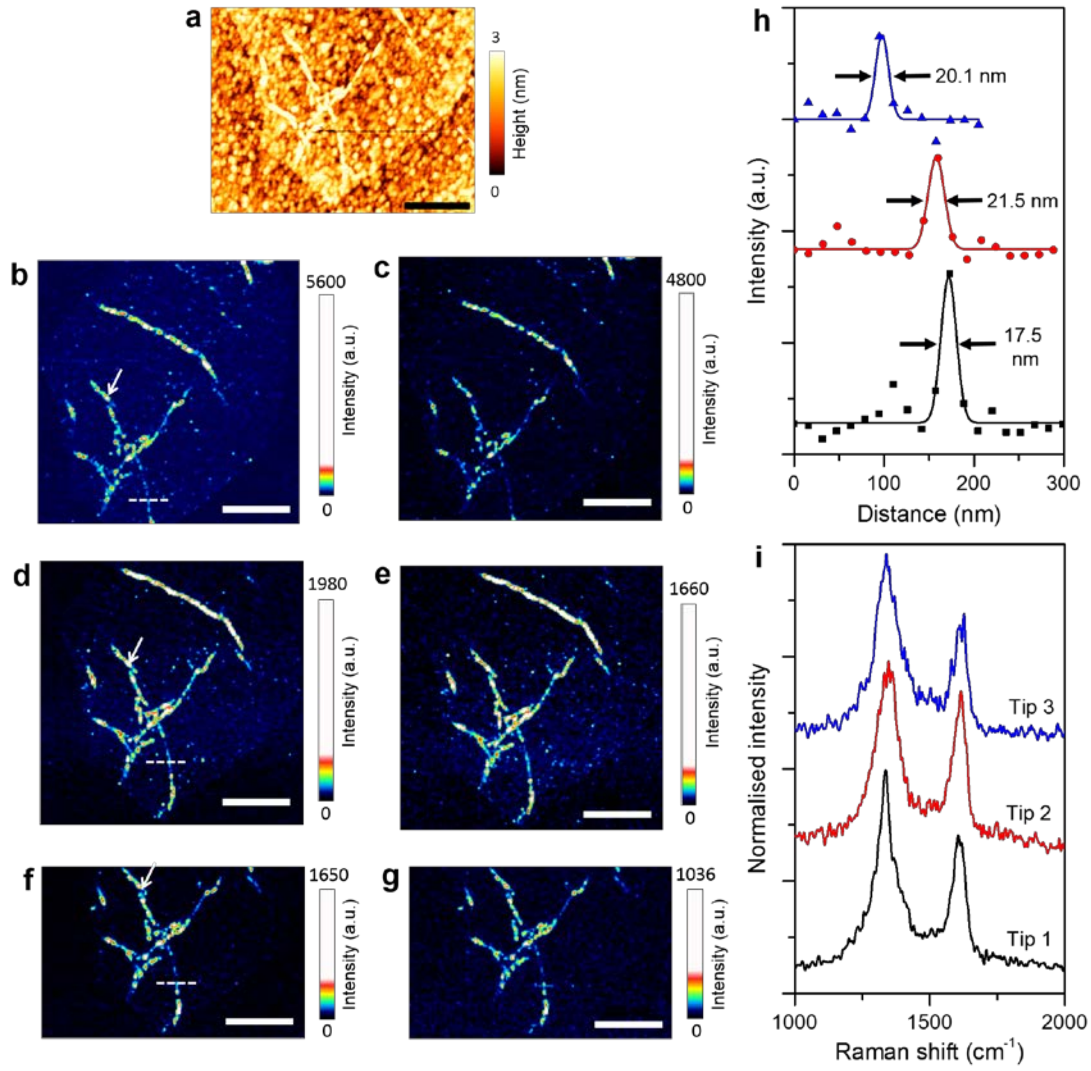

Supplementary Fig. 8 **a** AFM topography image of a few-layer GO-COOH flake obtained whilst TERS mapping. TERS maps of D and G band intensity obtained using **b**, **c** TERS tip 1; **d**, **e** TERS tip 2; **f**, **g** TERS tip 3, respectively. Number of pixels: 128 × 128 in **b** – **e**, 128 × 80 in **f** and **g**. Nominal step size: 15 nm. Integration time: 0.1 s. **h** Intensity profiles along the lines marked in **b** (blue), **d** (red) and **f** (black), along with the fitted Gaussian curves. **i** TERS spectra measured at the positions marked **b**, **d** and **f**, respectively. All scale bars: 500 nm.

obtained whilst TERS mapping. Supplementary Fig. 8b and 8c; 8d and 8e; 8f and 8g show the TERS maps of D and G band intensity obtained using TERS tips 1 – 3, respectively. These results demonstrate that high-resolution TERS mapping of the same GO-COOH flake can be reproducibly obtained using different TERS tips.

The spatial resolution of the TERS maps obtained using tips 1 – 3 is estimated from the FWHM of a Gaussian fit to the line profiles across a sharp feature marked in Supplementary Fig. 8b, 8d and 8f, respectively. A similar TERS spatial resolution of 20.1 nm, 21.5 nm and 17. 5 nm is obtained using TERS tips 1 – 3, respectively, as shown in Supplementary Fig. 8h. Note that a step size of 15 nm was used in these measurements, which is comparable to the spatial resolution to the TERS maps. However, a higher spatial resolution was obtained in the TERS map shown in Fig. 2, where a step size of 10 nm was used, indicating that the spatial resolution of TERS mapping could be limited by the step size.

Furthermore, three TERS spectra measured at the same position on the GO-COOH flake marked in Supplementary Fig. 8b, 8d and 8f are shown in Supplementary Fig. 8i. The three TERS tips measure almost the same TERS spectra at these locations, which indicates the high reproducibility of chemical imaging in these measurements.

Supplementary Note 5: Analysis of discontinuities within GO-COOH flake

The discontinuities in the GO-COOH flake were located by checking individual TERS spectra from the areas of low signal intensity in the TERS map shown in Fig. 3a. However, in this map, due to the very high signal intensity at certain locations, the low intensity areas are not clearly visible. Therefore, in order to highlight these areas, we remade Fig. 3a with a logarithmic scale, which is shown in Supplementary Fig. 9a. In this map, the very low intensity areas within the GO-COOH flake can be visualised more clearly. Averaged TERS

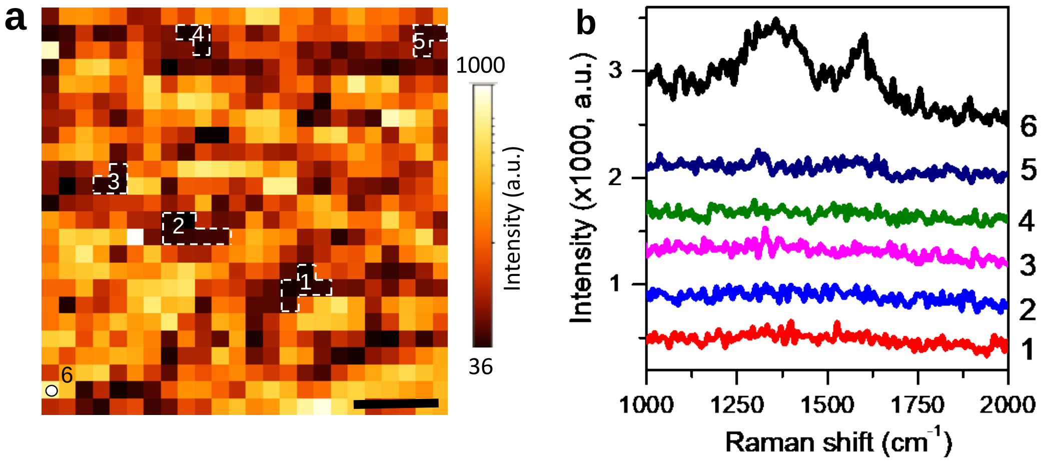

Supplementary Fig. 9 **a** Zoomed-in image of TERS D band intensity from the region marked with a dashed square in Fig. 2c, presented with a logarithmic intensity scale. Note that this image represents the same TERS map shown in Fig. 3a, which has a linear intensity scale. Scale bar: 50 nm. **b** Averaged TERS spectra from very low intensity areas marked as 1 – 5 in **a**. A TERS spectrum from a high signal intensity area at position 6 is also shown for comparison.

spectra from five very low intensity areas marked 1-5 in Supplementary Fig. 9a are shown in Supplementary Fig. 9b, where no D or G bands are visible indicating that these locations represent discontinuities within the GO-COOH lattice. The areas of the discontinuities were found to vary from 100 nm^2 to 600 nm^2 (1 – 6 pixels), which is of the order of step size used in the TERS map (10 nm) signifying that the discontinuities are very small in size. In the TERS map shown in Supplementary Fig. 9a, discontinuities were found to occupy 6.4 % of the total surface area.

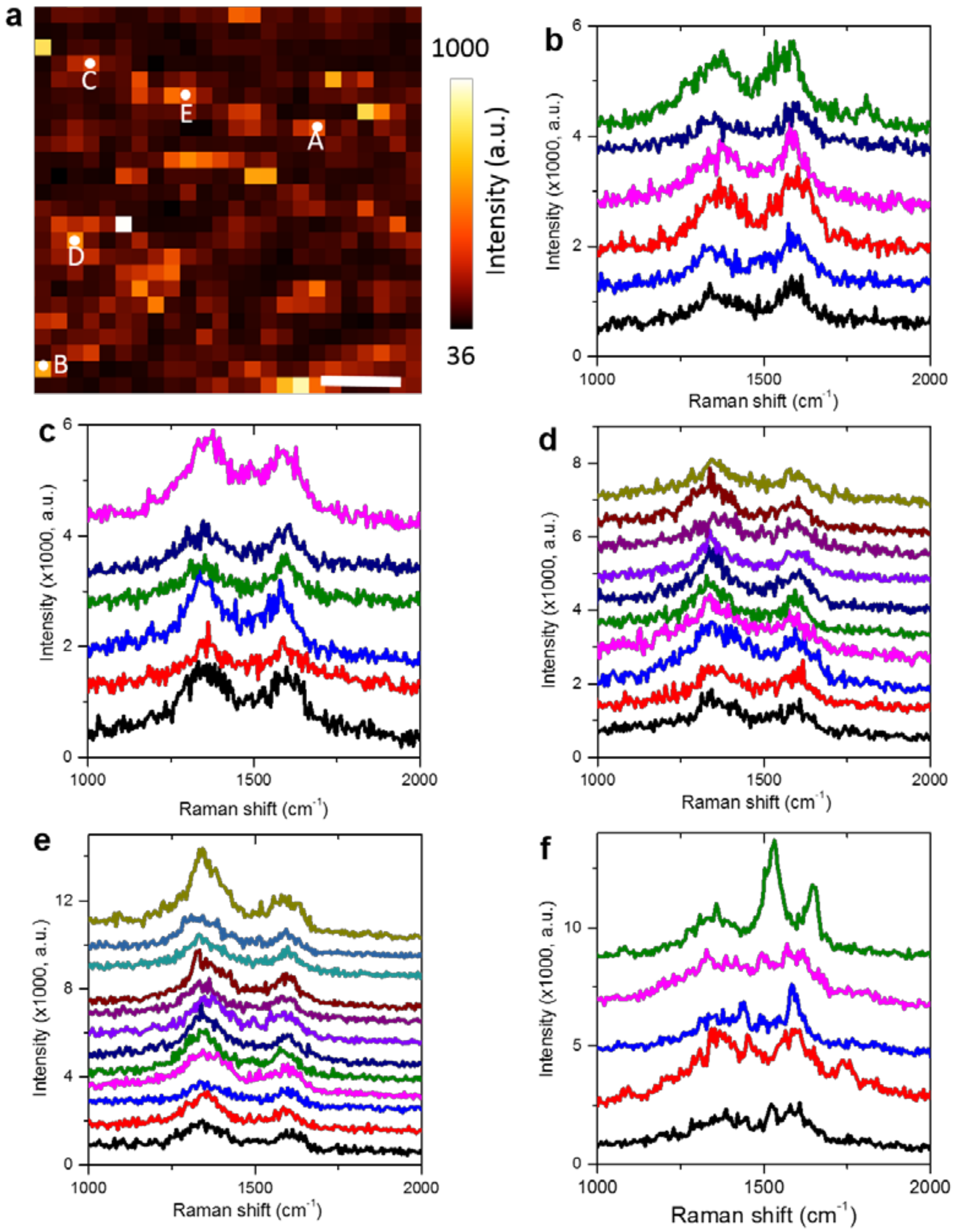

Supplementary Fig. 10 **a** Zoomed-in TERS map of the D band intensity from the region marked using a dashed square in Figure 2b. Scale bar: 50 nm. **b** – **f** TERS spectra similar to the spectra measured at positions A – E marked in **a**.

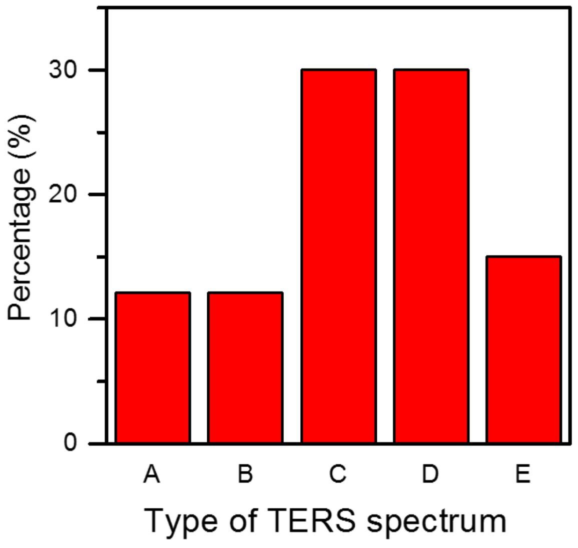

Supplementary Fig. 11 Histogram showing the statistical distribution of the type (A-E) of TERS spectra shown in Supplementary Fig.10b – 10f measured at 38 high intensity pixels of the TERS map in Supplementary Fig. 10a.

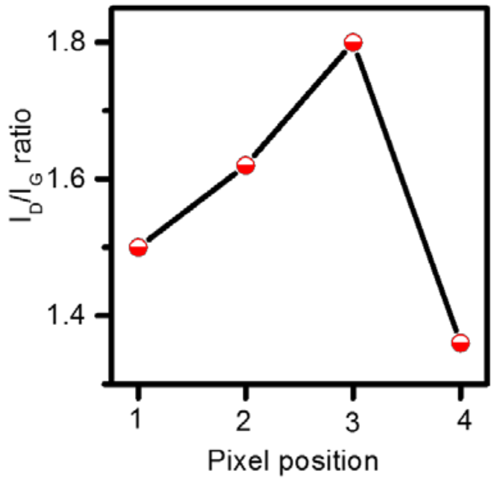

Supplementary Fig. 12 Variation of I_D/I_G ratio calculated from the TERS spectra at the pixels across position D shown in Fig. 3c. In this plot, pixel position 3 represents the position D marked in Fig. 3a.

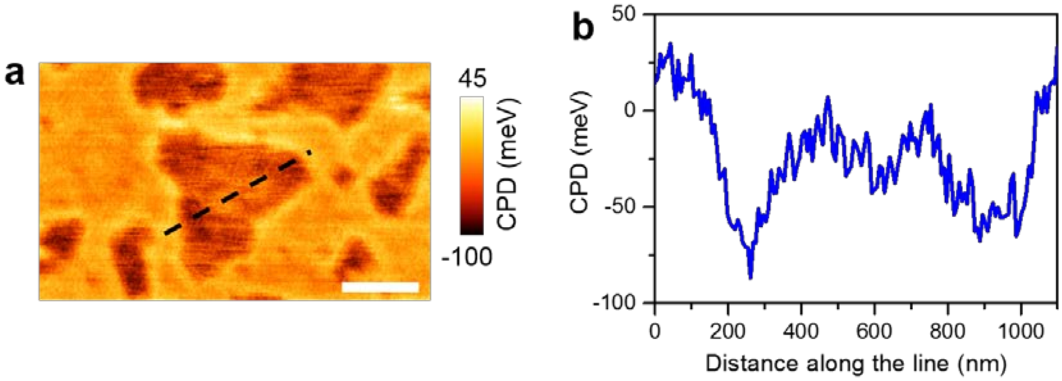

Supplementary Fig. 13 a CPD map measured in the region shown in Figure 4a. Scale bar: 500 nm. **b** CPD profile along the line marked in **a**.

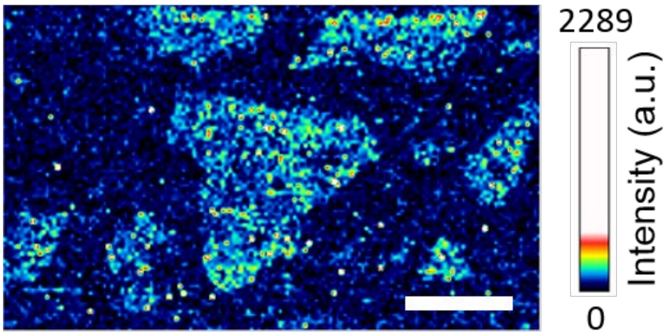

Supplementary Fig. 14 TERS map of G band (1590 cm^{-1}) intensity at measured in the region shown in Fig. 4a. Scale bar: 500 nm.

Supplementary Note 6: Correlation of CPD and I_D/I_G ratio

The spatial resolution of KPFM is generally much poorer than TERS[40]. For example, in our KPFM measurements although the step size of CPD map in Fig. 4b is as small as 2 nm, the spatial resolution of the image is ≈60 nm as shown in Supplementary Fig. 15b. The poor

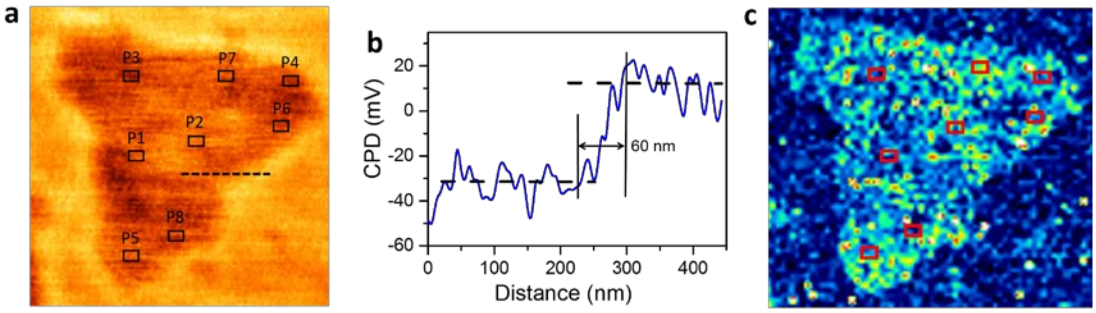

Supplementary Fig. 15 a Zoomed-in image of the CPD map shown in Fig. 4b. **b** Intensity profile along the black dashed line marked in **a** indicates a spatial resolution of ≈ 60 nm. **c** Zoomed-in image of the G band TERS map shown in Supplementary Fig. 14. Locations of 8 different regions selected for the comparison of average CPD and I_D/I_G ratio over the GO-COOH flake are marked in **a** and **c**.

spatial resolution of KPFM is also apparent from the relatively gradual variation of CPD inside the GO-COOH flake as shown in Supplementary Fig. 15b, where the CPD is observed to vary significantly over a length-scale of hundreds of nanometres. Therefore, in order to have a meaningful correlation of CPD variation with the local defect density, we calculated the I_D/I_G ratio from the TERS spectrum averaged over an area of 0.012 μm^2 (50 pixels) from eight different locations over the GO-COOH flake shown in Supplementary Fig. 15 and correlated it with the average CPD measured in the same areas.

Supplementary references

1. ACS material, https://www.Acsmaterial.Com/carboxyl-graphene-962.Html.
2. Lammel, T., Boisseaux, P., Fernández-Cruz, M.-L. & Navas, J. M. Internalization and cytotoxicity of graphene oxide and carboxyl graphene nanoplatelets in the human hepatocellular carcinoma cell line hep G2. *Part. Fibre Toxicol.* **10**, 27 (2013).
3. Rana, M., Balcioglu, M., Robertson, N. & Yigit, M. V. Nano-graphene oxide as a novel platform for monitoring the effect of LNA modification on nucleic acid interactions. *Analyst* **139**, 714-720 (2014).
4. Haubner, K. *et al.* The route to functional graphene oxide. *Chemphyschem* **11**, 2131-2139 (2010).
5. Acik, M. *et al.* The role of oxygen during thermal reduction of graphene oxide studied by infrared absorption spectroscopy. *J. Phys. Chem. C* **115**, 19761-19781 (2011).
6. Acik, M., Lee, G., Mattevi, C., Chhowalla, M., Cho, K. & Chabal, Y. J. Unusual infrared-absorption mechanism in thermally reduced graphene oxide. *Nat. Mater.* **9**, 840-845 (2010).
7. Chen, J., Wang, X. & Chen, T. Facile and green reduction of covalently pegylated nanographene oxide via a 'water-only' route for high-efficiency photothermal therapy. *Nanoscale Res. Lett.* **9**, 86 (2014).
8. Genin, F., Quiles, F. & Burneau, A. Infrared and Raman spectroscopic study of carboxylic acids in heavy water. *Phys. Chem. Chem. Phys.* **3**, 932-942 (2001).
9. Zhang, K., Kemp, K. C. & Chandra, V. Homogeneous anchoring of TiO_2 nanoparticles on graphene sheets for waste water treatment. *Mater. Lett.* **81**, 127-130 (2012).
10. Yang, D. *et al.* Chemical analysis of graphene oxide films after heat and chemical treatments by X-ray photoelectron and micro-Raman spectroscopy. *Carbon* **47**, 145-152 (2009).
11. Stadler, J., Schmid, T. & Zenobi, R. Nanoscale chemical imaging of single-layer graphene. *ACS Nano* **5**, 8442-8448 (2011).
12. Bonini, N., Lazzeri, M., Marzari, N. & Mauri, F. Phonon anharmonicities in graphite and graphene. *Phys. Rev. Lett.* **99**, 176802 (2007).
13. Jorio, A., Saito, R., Dresselhaus, G. & Dresselhaus, M. S. Raman spectroscopy in graphene related systems. 2011WILEY-VCHVerlag GmbH & Co (2011).
14. Smith, E. & Dent, G. Modern Raman spectroscopy– a practical approach. John Wiley & Sons, Chichester (2005).

15. Dong, J., Ozaki, Y. & Nakashima, K. Infrared, Raman, and near-infrared spectroscopic evidence for the coexistence of various hydrogen-bond forms in poly(acrylic acid). *Macromolecules* **30**, 1111-1117 (1997).
16. Neugebauer, U. *et al.* On the way to nanometer-sized information of the bacterial surface by tip-enhanced Raman spectroscopy. *ChemPhysChem* **7**, 1428-1430 (2006).
17. Dreyer, D. R., Park, S., Bielawski, C. W. & Ruoff, R. S. The chemistry of graphene oxide. *Chem. Soc. Rev.* **39**, 228-240 (2010).
18. Shao, F., Mueller, V., Zhang, Y., Schluter, A. D. & Zenobi, R. Nanoscale chemical imaging of interfacial monolayers by tip-enhanced Raman spectroscopy. *Ang. Chem. Int. Ed.* **56**, 9361-9366 (2017).
19. Jiang, N. *et al.* Nanoscale chemical imaging of a dynamic molecular phase boundary with ultrahigh vacuum tip-enhanced Raman spectroscopy. *Nano Lett.* **16**, 3898-3904 (2016).
20. Jiang, S. *et al.* Distinguishing adjacent molecules on a surface using plasmon-enhanced Raman scattering. *Nat. Nanotechnol.* **10**, 865-869 (2015).
21. Zhang, R. *et al.* Chemical mapping of a single molecule by plasmon-enhanced Raman scattering. *Nature* **498**, 82-86 (2013).
22. Yang, Z., Aizpurua, J. & Xu, H. Electromagnetic field enhancement in TERS configurations. *J. Raman Spectrosc.* **40**, 1343-1348 (2009).
23. Behr, N. & Raschke, M. B. Optical antenna properties of scanning probe tips: Plasmonic light scattering, tip-sample coupling, and near-field enhancement. *J. Phys. Chem. C* **112**, 3766-3773 (2008).
24. Pettinger, B., Ren, B., Picardi, G., Schuster, R. & Ertl, G. Tip-enhanced Raman spectroscopy (TERS) of malachite green isothiocyanate at au(111): Bleaching behavior under the influence of high electromagnetic fields. *J. Raman Spectrosc.* **36**, 541-550 (2005).
25. Robert, H. W. Confocal optical microscopy. *Rep. Prog. Phys.* **59**, 427 (1996).
26. Su, W., Kumar, N., Dai, N. & Roy, D. Nanoscale mapping of intrinsic defects in single-layer graphene using tip-enhanced Raman spectroscopy. *Chem. Commun.* **52**, 8227-8230 (2016).
27. Su, W., Kumar, N., Mignuzzi, S., Crain, J. & Roy, D. Nanoscale mapping of excitonic processes in single-layer MoS_2 using tip-enhanced photoluminescence microscopy. *Nanoscale* **8**, 10564-10569 (2016).
28. Park, K.-D., Khatib, O., Kravtsov, V., Clark, G., Xu, X. & Raschke, M. B. Hybrid tip-enhanced nanospectroscopy and nanoimaging of monolayer WSe_2 with local strain control. *Nano Lett.* **16**, 2621-2627 (2016).
29. Muller, V. *et al.* Structural characterization of a covalent monolayer sheet obtained by two-dimensional polymerization at an air/water interface. *Ang. Chem. Int. Ed.* **56**, 15262-15266 (2017).
30. Lee, Y., Park, S., Kim, H., Han, G. H., Lee, Y. H. & Kim, J. Characterization of the structural defects in CVD-grown monolayered MoS_2 using near-field photoluminescence imaging. *Nanoscale* **7**, 11909-11914 (2015).
31. Nozaki, J., Fukumura, M., Aoki, T., Maniwa, Y., Yomogida, Y. & Yanagi, K. Manipulation of local optical properties and structures in molybdenum-disulfide

monolayers using electric field-assisted nearfield techniques. *Sci. Rep.* **7**, 46004 (2017).

32. Saito, Y., Verma, P., Masui, K., Inouye, Y. & Kawata, S. Nano-scale analysis of graphene layers by tip-enhanced near-field Raman spectroscopy. *J. Raman Spectrosc.* **40**, 1434-1440 (2009).
33. Lee, Y. *et al.* Near-field spectral mapping of individual exciton complexes of monolayer WS_2 correlated with local defects and charge population. *Nanoscale* **9**, 2272-2278 (2017).
34. Park, K.-D., Raschke, M. B., Atkin, J. M., Lee, Y. H. & Jeong, M. S. Probing bilayer grain boundaries in large-area graphene with tip-enhanced Raman spectroscopy. *Adv. Mater.* **29**, 1603601 (2017).
35. Mignuzzi, S. *et al.* Probing individual point defects in graphene via near-field Raman scattering. *Nanoscale* **7**, 19413-19418 (2015).
36. He, Z. *et al.* Tip-enhanced Raman scattering on bulk MoS_2 substrate. *IEEE J. Sel. Top. Quant.* **23**, 4601006 (2017).
37. Ghislandi, M., Hoffmann, G. G., Tkalya, E., Xue, L. & De With, G. Tip-enhanced Raman spectroscopy and mapping of graphene sheets. *Appl. Spectrosc. Rev.* **47**, 371-381 (2012).
38. Su, W. & Roy, D. Visualizing graphene edges using tip-enhanced Raman spectroscopy. *J. Vac. Sci. Technol. B* **31**, 041808 (2013).
39. Bao, W. *et al.* Visualizing nanoscale excitonic relaxation properties of disordered edges and grain boundaries in monolayer molybdenum disulfide. *Nat. Commun.* **6**, 7993 (2015).
40. Zerweck, U., Loppacher, C., Otto, T., Grafstrom, S. & Eng, L. M. Accuracy and resolution limits of kelvin probe force microscopy. *Phys. Rev. B* **71**, 125424 (2005).
